# Adversarial training improves model interpretability in single-cell RNA-seq analysis

**DOI:** 10.1093/bioadv/vbad166

**Published:** 2023-11-23

**Authors:** Mehrshad Sadria, Anita Layton, Gary D Bader

**Affiliations:** Department of Applied Mathematics, University of Waterloo, Waterloo, Ontario N2L 3G1, Canada; Department of Applied Mathematics, University of Waterloo, Waterloo, Ontario N2L 3G1, Canada; Cheriton School of Computer Science, University of Waterloo, Waterloo, Ontario N2L 3G1, Canada; Department of Biology, University of Waterloo, Waterloo, Ontario N2L 3G1, Canada; School of Pharmacy, University of Waterloo, Waterloo, Ontario N2G 1C5, Canada; Department of Molecular Genetics, University of Toronto, Toronto, Ontario M5S 1A8, Canada; The Donnelly Centre, University of Toronto, Toronto, Ontario M5S 3E1, Canada; Department of Computer Science, University of Toronto, Toronto, Ontario M5S 2E4, Canada; The Lunenfeld-Tanenbaum Research Institute, Sinai Health System, Toronto, Ontario M5G 1X5, Canada; Princess Margaret Cancer Centre, University Health Network, Toronto, Ontario M5G 2M9, Canada

## Abstract

**Motivation:**

Predictive computational models must be accurate, robust, and interpretable to be considered reliable in important areas such as biology and medicine. A sufficiently robust model should not have its output affected significantly by a slight change in the input. Also, these models should be able to explain how a decision is made to support user trust in the results. Efforts have been made to improve the robustness and interpretability of predictive computational models independently; however, the interaction of robustness and interpretability is poorly understood.

**Results:**

As an example task, we explore the computational prediction of cell type based on single-cell RNA-seq data and show that it can be made more robust by adversarially training a deep learning model. Surprisingly, we find this also leads to improved model interpretability, as measured by identifying genes important for classification using a range of standard interpretability methods. Our results suggest that adversarial training may be generally useful to improve deep learning robustness and interpretability and that it should be evaluated on a range of tasks.

**Availability and implementation:**

Our Python implementation of all analysis in this publication can be found at: https://github.com/MehrshadSD/robustness-interpretability. The analysis was conducted using numPy 0.2.5, pandas 2.0.3, scanpy 1.9.3, tensorflow 2.10.0, matplotlib 3.7.1, seaborn 0.12.2, sklearn 1.1.1, shap 0.42.0, lime 0.2.0.1, matplotlib_venn 0.11.9.

## 1 Introduction

Deep learning is important in processing big biological data structures and has reshaped our ability to analyze large-scale datasets (Angermueller *et al.* 2016). However, the traditional black-box nature of deep neural networks (DNNs) remains a major obstacle to their wide adoption in applications where mechanistic insight is important (Azodi *et al.* 2020). A number of interpretability techniques have been developed to comprehend DNNs (Simonyan *et al.* 2013, Shrikumar *et al.* 2017, Selvaraju *et al.* 2020). For example, by using these techniques we can identify input features that significantly impact DNN output. Gradient calculations are also frequently employed to provide each input feature with a weighted significance score that reflects the impact of that feature on model predictions (Ancona *et al.* 2017). Deep learning models can also have problems with robustness, where their predictions are highly sensitive to small perturbations in input data (Chakraborty *et al.* 2021). These small perturbations can be specifically generated based on the trained model, in which case they are termed adversarial (Madry *et al.* 2017; Volpi *et al.* 2018). Adversarial training is a recent advance in deep learning, so far mainly applied to image and text inputs, that results in more robust and generalizable models (Bai *et al.* 2021; Yi *et al.* 2021). Adversarial training involves supplementing training data with generated adversarial instances during each training loop which leads to more robust models. For image applications, adversarially trained DNNs have been observed to yield loss gradients that are visually more interpretable than those from analogous models without adversarial training (Ross and Doshi-Velez 2018, Kim *et al.* 2019), but the interplay between robustness and interpretability in deep learning is poorly understood.

We study the relationship between robustness and interpretability in computational classification. We analyze, as a representative classification problem, the task of cell-type classification based on single-cell RNA-seq data. We choose this problem due to the abundance of training data available. Single-cell genomics technology has made it possible to measure gene expression profiles at single-cell resolution, providing an unprecedented opportunity to study the processes of multicellular organism growth, as well as disease and treatment response (Rood *et al.* 2022). To study these processes, cell types and states must be reliably identified to observe how they change over time (Ding *et al.* 2022). We also need to know key genes, such as gene expression markers or master regulators, that are useful for cell-type identification and mechanistic understanding of the underlying biological processes (Sadria *et al.* 2022; Sadria and Layton 2023). Machine learning models designed for automatic cell type identification from scRNA-seq data use various strategies, such as curated marker gene databases, reference expression correlation, and supervised classification (Alquicira-Hernandez *et al.* 2019, Tan and Cahan 2019, Lin *et al.* 2020, Yang *et al.* 2022).

In this work, we explore the effect of using adversarial training on the performance (accuracy, robustness, and interpretability) of deep learning models trained for cell-type classification using a range of simulated and real single-cell RNA sequencing data. We use several interpretability techniques to identify genes that are essential for cell type classification. Interestingly, we find that adversarial training increases both the robustness and interpretability of the resulting models and can be used to discover new biological insights, and suggest that this training approach will be useful to improve other classification problems in biology.

## 2 Methods

### 2.1 Interpretability

We used six machine learning model interpretability methods to compare our feature importance results:

Saliency map: The gradients with regard to inputs x are returned by the saliency map as feature importance score *S* (Simonyan *et al.* 2013):


$$s(x{)}_{i}=\partial M(x)/\partial x_{i}$$

by taking the first-order Taylor expansion of the neural network, *M*, as in:


$$M_{\left(x\right)}=\left(\frac{\partial M_{\left(x\right)}}{\partial x}\right)x+b$$

Activation maximization: A broad class of methods known as “activation maximization” looks for an input that maximizes the model response, generally using different gradient descent algorithms (Erhan *et al.* 2009). The idea is to generate an input that best represents the outcome by using:


$$X^{*}=\underset{x}{\mathrm{argmax}}f^{\left(l,k\right)}(x)$$

where *x* is the input and $X^{*}$ is the constructed input which maximizes the activation of a *k*th neuron in hidden layer l of the neural network *f*.

DeepLIFT: By back-propagating, the contribution of every neuron in the network to all features of the input, DeepLIFT decomposes the output prediction of a neural network on a particular input (Shrikumar *et al.* 2017):


$$\mathrm{DeepLIFT}{\left(x\right)}_{i}=\left(x_{i}-x_{i}^{\prime }\right)\times \frac{\partial^{g}M(x)}{\partial x_{i}},g\left(z_{t}\right)=\frac{f_{t}\left(z_{t}\right)-f_{t}\left(z_{t}^{\prime }\right)}{z_{t}-z_{t}^{\prime }}$$

DeepSHAP: DeepSHAP calculates the expectations of gradients by randomly selecting baseline data from the distribution and then uses that information to approximate SHAP (SHapley Additive exPlanations) values. Each input sample is first given white noise, and a random baseline is chosen from a predefined distribution. Next, a random point is chosen along the path between the base point and the input with noise, and the gradient of the outputs is computed with respect to the random point. To roughly estimate the expected values, *E*, of gradients, the technique is done numerous times. The final SHAP score is equal to:


$$E\left(\frac{\partial M(x)}{\partial x_{i}}\right)\times \left(x_{i}-x_{i}^{\prime }\right)$$

For further description of different interpretability methods implemented in the DeepSHAP package, see Lundberg and Lee (2017).

LIME: LIME is a surrogate method for explaining the predictions of machine learning models. It generates a new dataset of perturbed samples by making small changes to the original data, then training a simple, interpretable model on this new dataset. It then can be used to explain the prediction of the original model and compute feature importance scores that explain which features of the input had the most influence on the prediction. LIME is formulated as follows:


$$\Phi (x)={\mathrm{argmin}}_{g\in G}L\left(f,g,\pi_{x}\right)+\Omega (g)$$

A local model *g* from class *G* of interpretable models for an instance *x* is considered. To avoid having a complex model penalty term, Ω(*g*) is added. $\Pi_{x}$ denotes the neighborhood of *x* and *L* shows the loss between the complex and surrogate model in a defined local neighborhood $\Pi_{x}$.

### 2.2 Robustness

To generate adversarial data, the following methods are used, as found in Nicolae *et al.* (2018):

Fast gradient sign method (FGSM): Adding practically unnoticeable noise to the input data is a strategy to create an adversarial attack. A common attack method is FGSM (Goodfellow *et al.* 2014). FGSM adds noise in the direction of the gradient which reduces the accuracy of the prediction. For an attack level *ϵ*, FGSM sets:


$$x_{\mathrm{adv}}=x+\epsilon \mathrm{sign}\left(\nabla_{x}L(f(x),y)\right)$$

The attack level is chosen to be sufficiently small so as to be undetectable. The optimal *ε* value depends on the characteristics of the data and specific task.

Projected gradient descent (PGD): PGD is an upgraded version of FGSM that employs several iterations (Madry *et al.* 2017). In the equation below, Proj denotes the projection operator, which constrains the input to positions set by a predefined perturbation range. ε is the step size with a positive value. PGD works as follows:


$$x_{N+1}^{\mathrm{adv}}={\mathrm{Proj}}_{x,\epsilon }\left\{x_{N}^{\mathrm{adv}}+\alpha \mathrm{sign}\left(\nabla_{x}J\left(x_{N}^{\mathrm{adv}},y_{\text{true}\;}\right)\right)\right\}$$

### 2.3 Data processing and model training

All scRNA-seq gene expression data were scaled, centered, and log-normalized. The top 2500 highly variable genes are selected from the ones that have at least 30 expressed counts. We train a cell-type classification model (neural network architecture in Supplementary Table S1). For the training process after the preprocessing steps, we divided the data into 80% for training and 20% for testing. For classification tasks, we applied categorical cross-entropy loss to all datasets except the Allen Brain Map, where we employed sparse categorical cross-entropy loss, following the original authors (Le *et al.* 2022).

### 2.4 Gene set enrichment analysis

Differential gene expression analysis, used as a baseline, was computed using the Wilcoxon rank-sum test and Benjamini–Hochberg multiple testing correction. Genes with a corrected *P*-value below .05 were regarded as statistically significant and selected for further use. Gene set enrichment analysis was performed using the gProfiler web platform (Raudvere *et al.* 2019). We used Benjamini–Hochberg false discovery rate (FDR) values for the Gene Ontology (GO) terms.

### 2.5 Adversarial attack on established cell annotation approaches

For cell classification using the lung reference data, we use the Python package called pySingleCellNet. The reference data have 2500 cells and 16 543 genes. We sample 200 cells from each cell type to have a balanced training dataset, and the rest is used to evaluate the performance of the model (the authors of SingleCellNet recommended 50 cells as the minimum to achieve an accurate classifier). In addition, we select genes so that both the query data and reference data are limited to a common set of genes before training the classifier. All model hyperparameters are set to the default values suggested by the authors (Tan and Cahan 2019).

### 2.6 For visualization, reduced dimension data

Single cell transcriptomics data and clusters were visualized using the Python package “UMAP”.

## 3 Results

### 3.1 Adversarial training improves robustness for cell type classification

**Figure 1.**
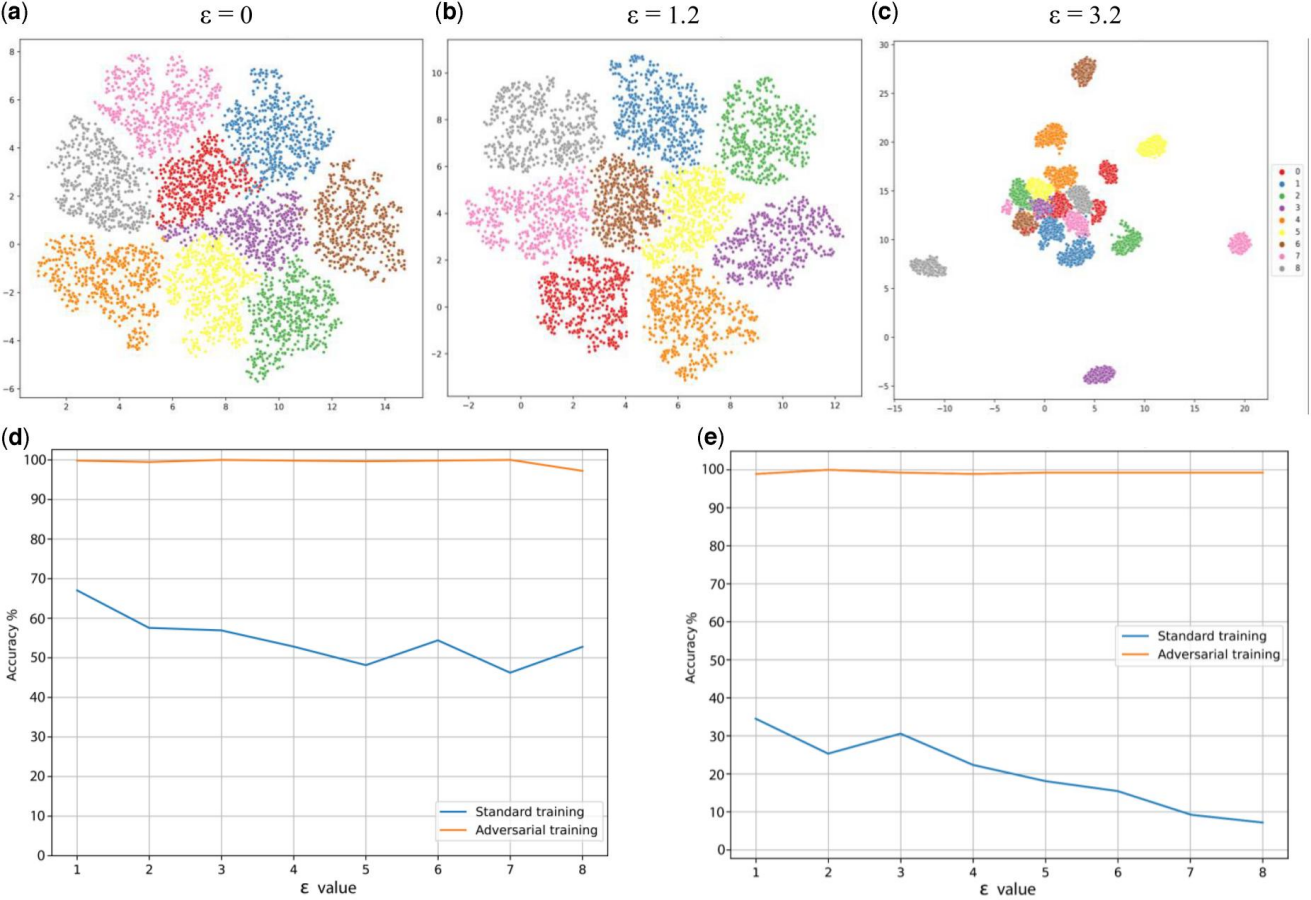
Effects of adversarial attack on data structure and model accuracy. UMAP plots (a) of original data, (b) with an adversarial attack using FGSM with an epsilon value of 1.2, which preserves overall data structure, and (c) with an adversarial attack with a larger epsilon value of 3.2, which substantially changes data structure. Effect on model accuracy following FGSM (d) and PGD attacks (e). Adversarial attacks reduce model accuracy substantially (blue lines), but with appropriate adversarial training, high accuracy can be achieved (orange lines).

To explore model robustness using adversarial training, we select single-cell classification as an example task. A multi-layer perceptron architecture (detailed architecture in Supplementary Table S1) is selected to implement this task. Single-cell RNA-seq data, represented as a cell by gene matrix, with a given set of ground truth cell classes are used as input. Initially, we use simulated data generated by SERGIO (Dibaeinia and Sinha 2020) to ensure we work with a concrete ground truth data set. SERGIO enables users to specify the number of cell types to be simulated, given a simulated gene regulatory network (Supplementary Fig. S1). We use SERGIO to generate a gene expression matrix of 2700 cells and 1200 genes with nine cell types. The gene-by-cell matrix and cell type labels are used to train a classifier with a hyperparameter search on the number of layers and nodes. The classifier achieves an accuracy of 98.2% on the simulated data. We then use two established methods to generate adversarial data which are required for adversarial training: PGD and FGSM (Goodfellow *et al.* 2014, Madry *et al.* 2017). These take the trained model and introduce noise in the input data in the direction of the model gradient that has the greatest impact on the model’s accuracy. However, we must tune the amount of noise, *ε*, in this procedure, as adding too much or not enough noise will not result in useful adversarial data for training. In typical applications such as computer vision, the value of *ε* is well-established for different methods (Goodfellow *et al.* 2014, Madry *et al.* 2017;). However, tabular data, like scRNA-seq, have received less attention in identifying the appropriate *ε* value. A good *ε* value is one that causes a noticeable reduction in the classifier's accuracy while not substantially interfering with the structure of the input data. Therefore, we vary the *ε* value, while generating adversarial training data using PGD and FGSM with our simulated gene expression data (input) and evaluate the results. The newly generated adversarial data are combined with the original data to create an adversarial training data set with 5400 cells, comprising 2700 adversarial cells and 2700 original unperturbed cells (Supplementary Fig. S2a). We evaluate the *ε* value in two ways: classification performance of the original model on the adversarial training data set and manual evaluation of the stability of the global data structure using UMAP visualization (Fig. 1). Values of *ε* between 1 and 1.2 significantly decrease classifier accuracy while maintaining global structure, when the model is subjected to FGSM perturbation (Fig. 1b and d blue line). However, larger *ε* values (e.g. 3.2) cause the global structure to degrade, indicating that the selected value is too large (Fig. 1c). We next test the effect of adversarial training using our established *ε* value, comparing model accuracy and F1 score without adversarial training (Fig. 1d and e and Supplementary Fig. 2c and d blue lines) to accuracy and F1 score with adversarial training (Fig. 1d and e and Supplementary Figure 2c and d orange lines). We find that training with adversarial data using both FGSM and PGD significantly strengthens the model’s robustness, raising accuracy from as low as 30% with standard training to almost 100% accuracy with adversarial training.

Next, we assess the impact of adversarial training on class-imbalanced data, specifically evaluating its effect on the robustness of smaller cell types. To achieve this, we remove 75%–85% of cells from three different cell types (out of nine) in our simulated gene expression data. We then train a neural network using this reduced dataset and generate adversarial data, which, when integrated with the original data, forms the adversarial training dataset. Subsequent retraining of the model using this adversarial training data again leads to a notable increase in model robustness across all cell types, regardless of the cell number within each type (Supplementary Fig. S2b) which demonstrates the efficacy of adversarial training in enhancing the robustness of models trained on scRNAseq data with cell types of varying counts, even in unbalanced data.

### 3.2 Adversarial training improves model interpretability

While adversarial training fortifies a neural network against adversarial perturbations and increases the robustness of the model, its effects on interpretability are not well studied. Many DNN interpretability methods are based on analyzing weights and gradient loss; therefore, adversarial training may affect model interpretability (Ancona *et al.* 2017). To study this relationship, we apply an adversarially trained neural network to classify cell-types with our SERGIO-simulated scRNA-seq data generated to include 65 predefined key genes and then identify significant genes for each cell type using six different DNN interpretability methods (saliency maps (Simonyan *et al.* 2013), activation maximization (Erhan *et al.* 2009), Local Interpretable Model-agnostic Explanations (LIME) (Ribeiro *et al.* 2016), and three variants of SHapley Additive exPlanations (SHAP) (Lundberg and Lee 2017): gradient explainer, deep explainer, and kernel explainer). All these methods use a local linear approximation to identify important features of a model. However, the loss functions and local neighborhood definition differ among these methods, which often results in discrepancies and differences in their results.

**Figure 2.**
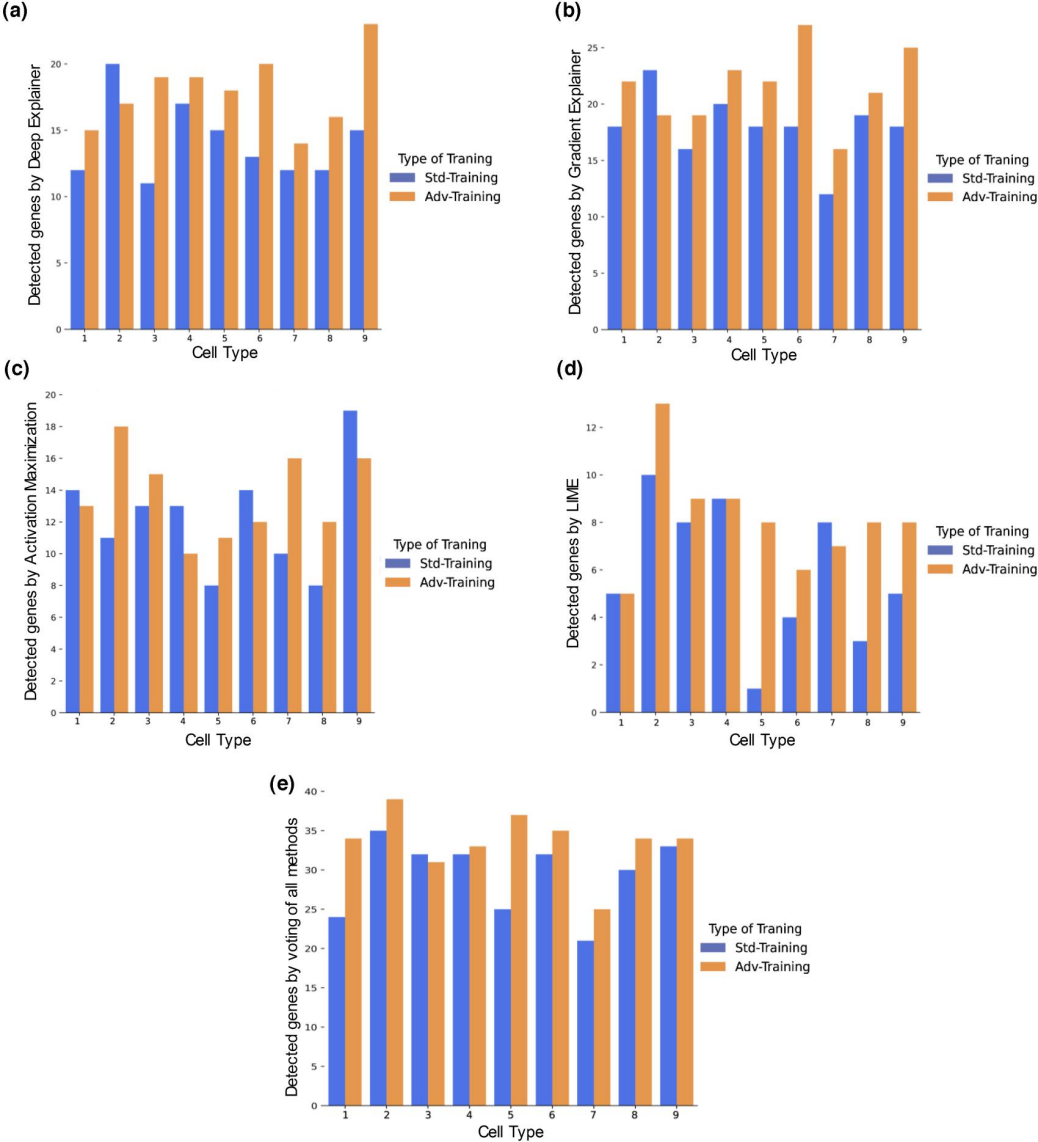
The effect of adversarial training on the model’s interpretability. (a) The results of using the Deep Explainer, (b) gradient explainer, (c) activation maximization, (d) LIME, and (e) the number of detected key genes using an aggregate result of all methods before and after adversarial training.

To measure the effect of adversarial training on the accuracy of the interpretability methods, we compute the number of predefined key genes correctly detected by our interpretability methods for each cell type with and without adversarial training. For most of the cell types, an adversarially trained neural network detects more key genes than the non-adversarially trained model (Fig. 2a–d and Supplementary Fig. 3). These results indicate that, in general, adversarial training improves the accuracy of the interpretability methods. As expected by the no-free-lunch theorem for explanation methods, there is substantial variability in the performance of various interpretability methods (Supplementary Fig. 4), which depends on the characteristics of the data and the local context (Han *et al.* 2022). Based on this observation, we use a voting mechanism that considers the aggregate of significant genes recognized by all six interpretability methods and calculates a “consensus importance score”. This score denotes the number of interpretability methods that identify a specific gene as one of the top N genes for a given cell type and is used as a primary output for our method. Specifically, we consider the top 20 most important genes identified by each method and rank the genes based on the majority vote. Results in Fig. 2e show that adversarial training increases the number of correctly identified key genes in almost all cell types for this majority vote approach. These results suggest that adversarial training not only can improve model robustness but also its interpretability, as measured by the number of correctly identified key genes.

### 3.3 Using adversarial training can help discover key genes and pathways in single-cell RNA-seq data

**Figure 3.**
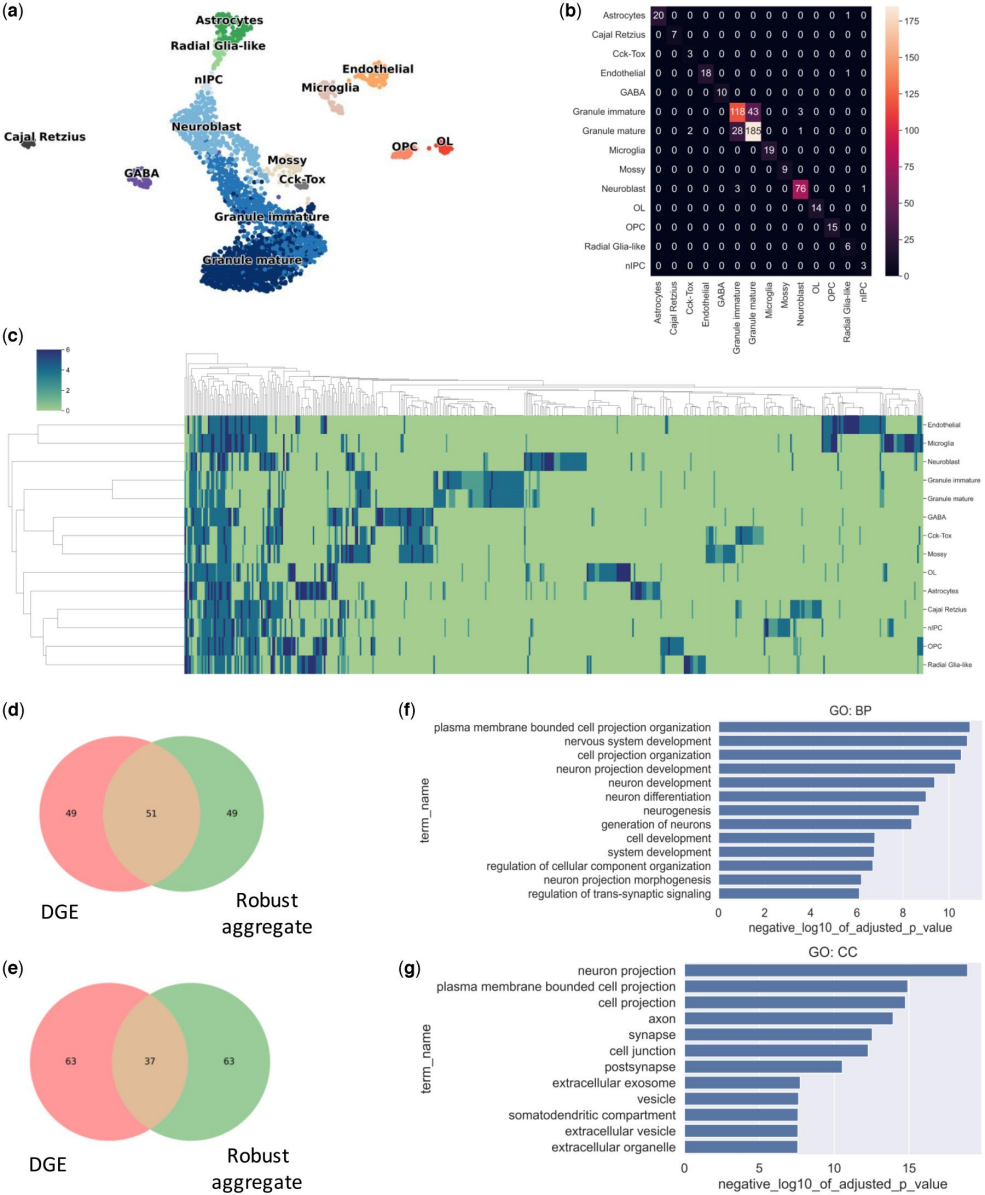
Applying adversarial training to identify important genes in mouse hippocampus development. (a) UMAP visualization of mouse hippocampus development data (14 cell types). (b) The confusion matrix of the classifier. (c) The consensus importance scores of genes determined by applying multiple interpretability methods visualized as a clustered heatmap. (d, e) Venn diagrams show the comparison of the number of genes detected by differential gene expression and adversarial training based on importance prediction for astrocyte and endothelial cells, respectively. (f, g) The pathway and cellular component gene set enrichment analysis, respectively, of the predicted important genes. DGE, differential gene expression.

To establish the applicability of the adversarial training in real biological data, we analyze a mouse hippocampus development scRNA-seq dataset of 18 231 cells, 14 cell types, and 3,001 genes (Hochgerner *et al.* 2018) (Fig. 3a). We first train our non-adversarially-trained DNN classifier to predict known cell types. The classifier performs well for the majority of cell types, except for the distinction between mature and immature granulocytes (Fig. 3b). When the model is adversarially attacked using PGD, its accuracy decreases significantly (Supplementary Fig. S5). To increase its robustness, we adversarially train the classifier, apply all six interpretability methods to the resulting model to identify key genes for each cell type (Supplementary Fig. S6), and compute the “consensus importance score” for each gene-cell type pair (*N* = top 150 genes). We confirm that adversarial training of the model using both FGSM and PGD significantly improves its robustness (Supplementary Fig. S5a and b). We then cluster the genes according to their consensus importance scores and visualize the results as a heatmap (Fig. 3c). Notably, genes located on the left side of the heatmap are selected as important for classifying the majority of cell types. In contrast, genes located on the right side of the heatmap are important for classifying specific types. Table 1 shows the top predicted important genes based on the consensus importance scores computed for each cell type. Most genes in this table (50 of 59) are known to play a key role in that specific cell type or in neuronal development in general (Song *et al.* 2013, Kato *et al.* 2020, Magnusson *et al.* 2020, Todd and Hardingham 2020, Fatima *et al.* 2021, Supplementary Table S2).

**Table 1.** Genes predicted as important (high consensus importance score) for each cell-type for the mouse hippocampus dataset.

Cell type	Predicted important genes
Astrocytes	Gad2, Dbi, Fabp7, Igfbpl1, Cplx2
Cajal Retzius	Dbi, Itm2b, Tmsb10, Cplx2, **Atp5g1**
Cck_Tox	Gad1, Igfbpl1, **Scg5**, **Ppfia2**, **Lamtor2**
Endothelial	Sept4, Klf2, Igfbpl1, Snca, Cnih2
GABA	Nrgn, Npy, Itm2a, Cst3, Calm2
Granule immature	Ncdn, **Lin7b**, Camk4, Snca, **Tspan3**
Granule mature	**Dmtn**, **Rbm25**, **Rap1b**, Cpe, C1ql3
Microglia	Nrgn, Hexb, P2ry12, Cnih2, Cplx2
Mossy	B2m, Trappc4, Camk4, **Cntn1**, **Mycbp2**
Neuroblast	**Dnaja1**, **Arpc1a**, Cfdp1, **Nell2, Prdx2**
OL	Camk2a, Mllt11, Sept7, Tubb4a, **Fez1**
OPC	Stmn1, Stmn4, Fabp7, Scrg1, Golga7
Radial Glia-like	Dbi, Zbtb20, Slc1a2, Scrg1, Ncdn
nIPC	Camk2b, Bcl11b, **Aplp1**, Arl6ip1, **Hmgn3**

The bolded genes are the ones that were not detected by DGE but were detected by consensus importance score.

To further validate the important genes we predict, we perform pathway and cellular location enrichment analysis on the 15 most important predicted genes from each cell type (total = 210). This analysis shows that pathways related to nervous system development and neurogenesis are significantly enriched in these genes (Fig. 3f), as well as brain-related cellular compartments such as neuron projection, synapse, and axon (Fig. 3g), as expected. As a control, we compare our predicted important genes to the list of differentially expressed genes, selected as a standard method to identify important genes. We compute for astrocytes and endothelial cells the top 100 predicted important genes by our model and by differential gene expression analysis. For astrocytes, half of the top genes are shared between our method and differential gene expression analysis; for endothelial cells, about one-third (see Fig. 3d and e). Examining the top five genes from each cell type, we observe that 50 of 59 genes are only found by our consensus importance score, and most of these are important for the brain (Supplementary Table S2).

**Figure 4.**
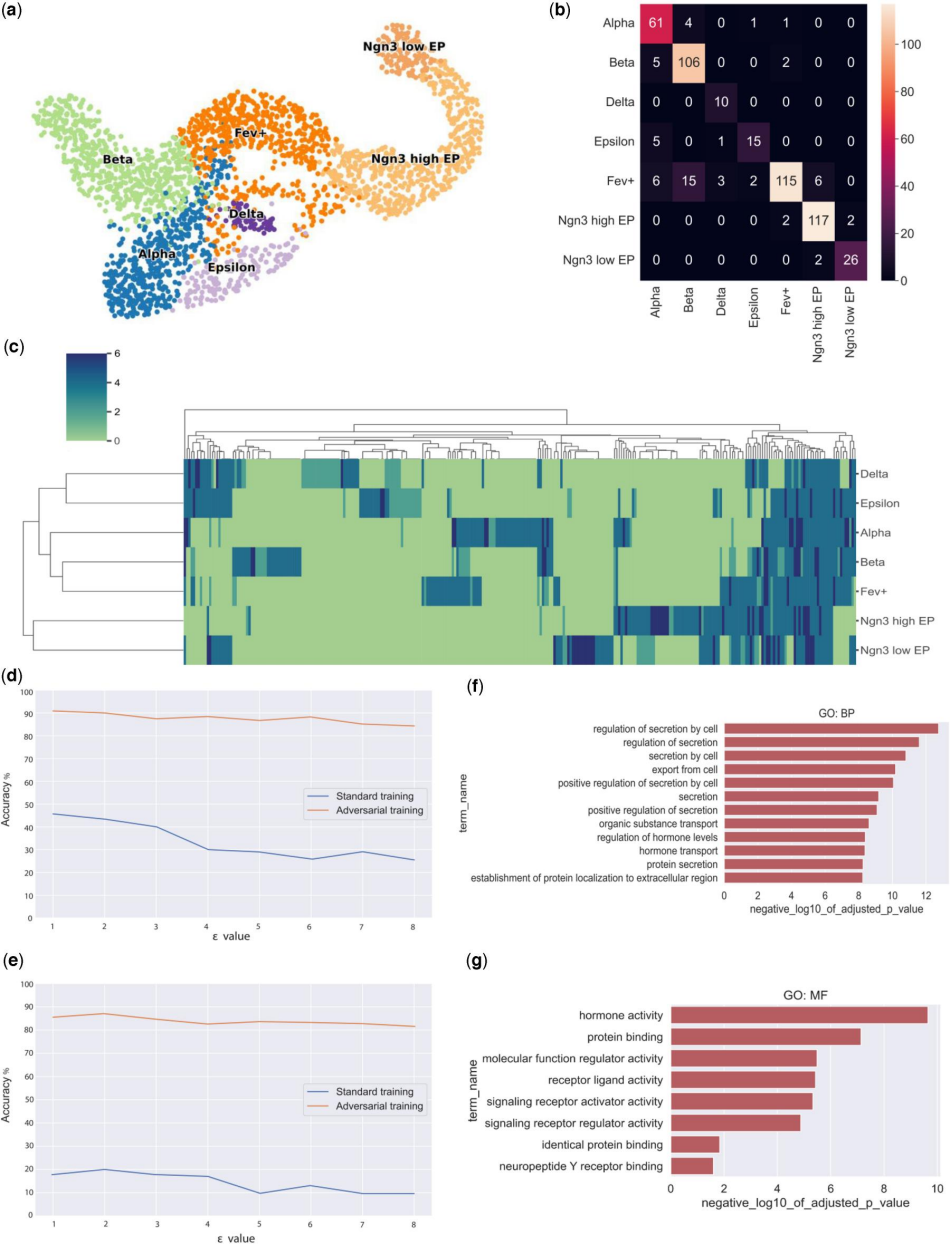
Representation of the mouse pancreas data after applying interpretability methods to an adversarially trained classifier. (a) The UMAP visualization of the data, highlighting the clustering of different cell types. (b) The confusion matrix of the classifier, shows the model's performance in cell-type classification. (c) The heatmap of the consensus importance scores was determined by applying multiple interpretability methods for each gene cell-type pair. (d, e) The accuracy of the model before and after adversarial training using PGD and FGSM methods, respectively. Lastly, (f) and (g) showcase the gene set enrichment analysis of the crucial genes identified by the consensus importance scores, providing a deeper understanding of the underlying biological process and molecular function.

As an additional test, we repeat our analysis on a mouse pancreas scRNA-seq dataset collected from the 15.5th day of embryonic development (Bastidas-Ponce et al. 2019) with 2531 cells clustered in seven cell types (Fig. 4). By using the adversarially trained model using PGD and FGSM, we are able to increase the model robustness (Fig. 4d and e). We again see that many of the important genes predicted by our method using the consensus importance score (Fig. 4c) play a key role in pancreas development (Table 2) (Liu *et al.* 2011, Muraro *et al.* 2016, Byrnes *et al.* 2018, Millership *et al.* 2018, Sakata *et al.* 2019, Fujita *et al.* 2021, Bosi *et al.* 2022). Moreover, we observe substantial enrichment of pathways and cellular components specific to pancreas-related functions within the gene sets associated with each cell type. These functions include key aspects such as the regulation of cell secretion, hormone activity, hormone transport, and pancreas development (Fig. 4f and g).

**Table 2.** Gene candidates detected for each cell-type using consensus scores for the developing mouse pancreas data.

Cell type	Predicted important genes
Alpha	Gcg, Arx, Tmem27, Iapp, Peg10, Tmsb15l, Btg2, Isl1, Txnip, Pcsk2
Beta	Pdx1, Gcg, Iapp, Gng12, Btg2, Pyy, Ghrl, Ins2, Fuca1, Nnat
Delta	Tuba1a, Sst, Tmsb4x, Hhex, Arg1, Sox9, Gm1673, Ins2, Pdx1, Gcg
Epsilon	Iapp, Gpx3, Chgb, Pdx1, Cck, Hspa5, Ghrl, Mboat4, Rrbp1, Tuba1a
Fev+	Nnat, Gng12, Gpx3, Ins2, Ppp1r1a, Sult2b1, Fev, Chgb, Cck, Hspa5
Ngn3 high EP	Tuba1a, Pdx1, Tspan7, Cryba2, Chga, Tm4sf4, Btg2, Tmsb4x, Txnip, Pclo
Ngn3 low EP	Pdx1, Cryba2, Chga, Txnip, Gng12, Chgb, Rbp4, Spp1, Cpe, Akr1c19

The table includes columns for the cell-type and the detected gene candidates.

### 3.4 Adversarial attack on established cell annotation approaches

**Figure 5.**
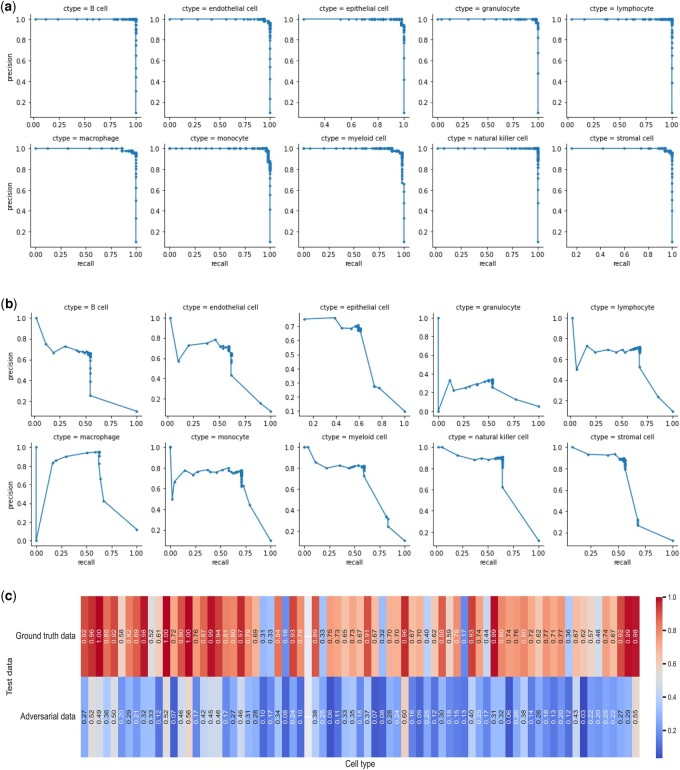
Effect of FGSM adversarial attacks on a trained single cell classification model using reference data. (a) Precision and recall values for different cell types using the SingleCellNet trained model applied to the ground truth mouse lung query data. (b) Precision and recall plots for various cell types using the same trained model, this time using the adversarial mouse lung query data. (c) F1 scores of a cell-type classifier trained on Allen Brain Map and tested on ground truth data (first row) and adversarial data (second row).

We tested adversarial attack of cell type classification using scRNA-seq data from two published cell classification studies. We selected studies that used gradient-based machine learning methods (required for adversarial training), written in Python, and with open, easily reproducible data to enable us to retrain the models in our experiments. First, we incorporated the well-annotated lung reference map to train a modified version of the cell annotation method SingleCellNet (Tan and Cahan 2019). The original model used a random forest, which has a non-continuous step function and is not compatible with gradient-based techniques like FGSM and PGD. Consequently, we changed the random forest model to a support vector machine. We then used an independent mouse lung dataset to evaluate the mapping performance and the influence of adversarial attack on the accuracy of cell-type classification. The precision-recall results show that adversarial attack significantly affects the performance of the trained model (Fig. 5a and b), in agreement with our original results. We also investigate the effect of adversarial attacks on an alternative cell-type classification method trained using the large Allen Brain Map dataset (Le *et al.* 2022). This dataset consists of 15 603 cells and 13 944 genes, clustered into 75 distinct cell types, including 45 inhibitory neurons, 24 excitatory neurons, and 6 non-neuronal types. We divide the data into training (60%), validation (20%), and test (20%) datasets and train a multi-layer perceptron for cell-type classification using the training set, using the published architecture and hyperparameters of the original authors (Le *et al.* 2022). The trained model achieves a high F1 score for each cell type (Fig. 5c first row). However, following the FGSM attack on the trained neural network, the model's F1 score drops precipitously across all cell types (Fig. 5c second row). Together, these results highlight the vulnerability of existing cell classification approaches to adversarial attacks.

## 4 Discussion

It is essential for machine learning models used in analyzing biological and clinical data to be accurate and robust. Interpretability is also crucial as it provides insight into the underlying processes and is useful for the design of new interventions. Here, we first show how adversarial training is effective for making deep learning models trained on single-cell RNA-seq data more robust using simulated and real data. Second, we show that there is a connection between the robustness and interpretability of these deep learning models. Deep learning interpretability methods can identify more significant genes (e.g. markers and key regulators) in cell-type classification tasks when applied to adversarially trained models than the analogous ones without such training. We speculate that adversarial training improves interpretability by constraining gradients to be closer to the data manifold, as seen in other domains (Kim *et al.* 2019). We also find that the genes we identify from interpreting our deep learning model are not simply those identified by standard differential expression analysis (selected as a baseline method). Presumably, the deep learning model is identifying additional important factors in the data to identify these genes and further research is needed to gain a deeper understanding of how this selection process occurs.

Our findings shed light on the important connection between model interpretability and robustness in machine learning. Adversarial training also fortifies a deep learning model, which can be useful for future clinical and health applications, such as diagnostic or prognostic gene expression biomarkers or patient classification, that need to be robust against adversarial attacks (Rood *et al.* 2022). We hope our study will encourage researchers to consider adversarial training and the robustness-interpretability relationship in future deep learning research in biology and medicine.

## 5 Limitations of study

Challenges related to false-positive and false-negative marker genes can impact the precision of interpretability methods. False-positive markers are genes inaccurately identified as significant contributors, which can arise from factors such as data noise, model complexity, or correlations among features. Conversely, false-negative markers, essential genes overlooked by explanation methods, may result from subtle influences or intricate interactions. These considerations highlight the cautious application and validation of these models, particularly when using complex datasets, and also the importance of employing simulated data with known ground truth, such as the ones we used in this study, to serve as a valuable tool for validation purposes. Adversarial training adds time to model development and may be time consuming for large models, depending on the adversarial training method used.

## Supplementary Material

vbad166_Supplementary_Data

## Data Availability

Simulated data are generated by SERGIO (Dibaeinia and Sinha 2020) and can be found at https://github.com/PayamDiba/SERGIO. We downloaded a preprocessed version of the “Development of the murine pancreas” dataset (Bastidas-Ponce et al. 2019) from https://cellrank.readthedocs.io/en/stable/index.html. We downloaded a preprocessed version of the “Dentate Gyrus neurogenesis” data (National Center for Biotechnology Information’s Gene Expression Omnibus repository, accession number GSE95753) from https://scvelo.readthedocs.io/en/stable/. We downloaded the reference mouse lung data from the Tabula Muris dataset (Tabula Muris Consortium et al. 2018) reference and query data were downloaded from https://github.com/pcahan1/PySingleCellNet. The human brain snRNA-seq dataset was downloaded from the Allen Brain Map https://portal.brain-map.org/atlases-and-data/rnaseq.
