# Supplementary material for: Adversarial training improves model interpretability in single-cell RNA-seq analysis
Source: Bioinform Adv. 2023 Nov 23;3(1):vbad166. doi: 10.1093/bioadv/vbad166 (PMC10719216; doi:10.1093/bioadv/vbad166)
Supplement: vbad166_Supplementary_Data [file vbad166_supplementary_data.zip › Robustness_Supplement_file.docx]

**
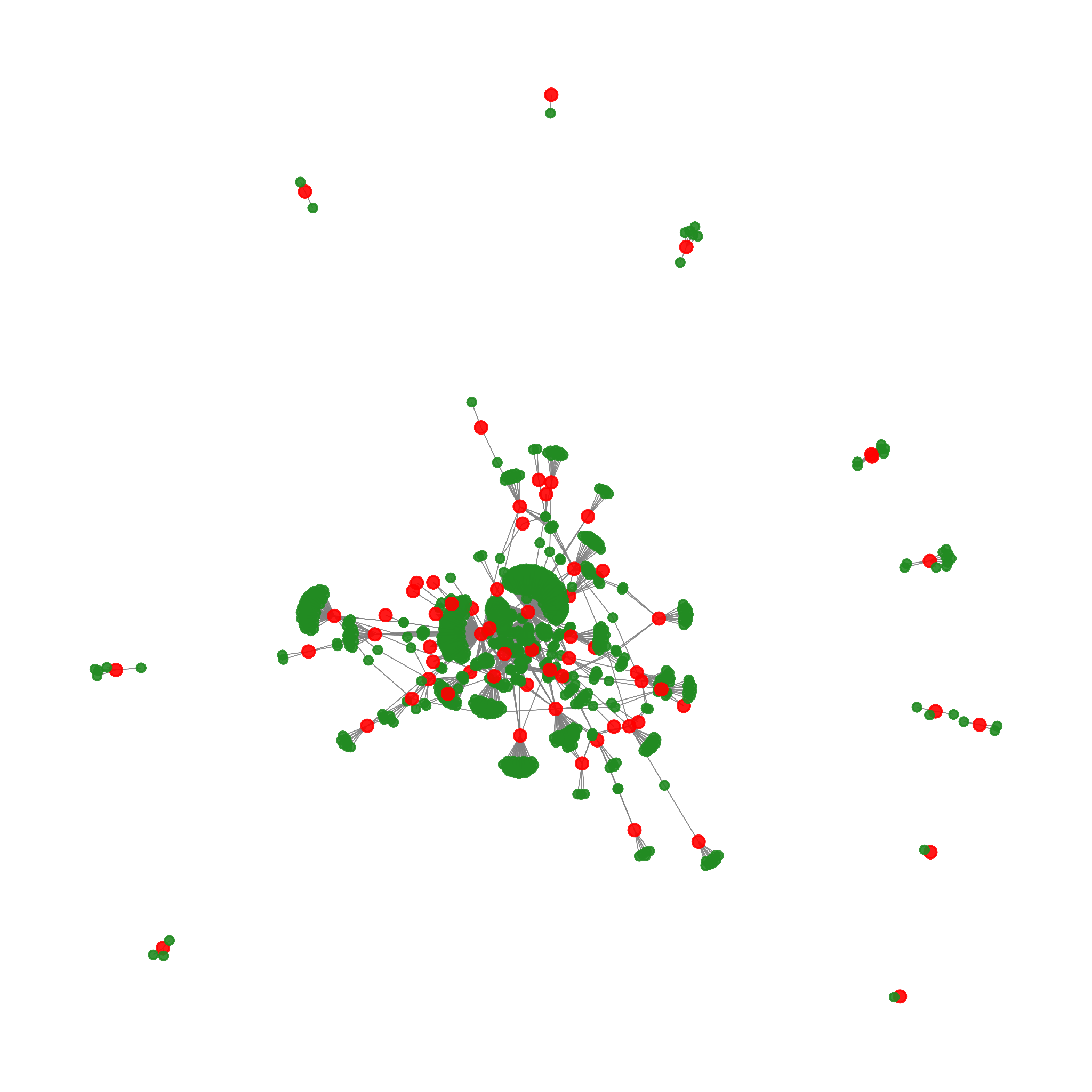
**

**Supplementary Figure 1 – Schematic gene regulatory network of scRNA-seq data generated by SERGIO.**

The gene regulatory network by SERGIO depicts the interaction between genes within the system. Nodes in red denote key genes (master regulators), while nodes in green represent genes, whose production rates are influenced by their respective regulators.


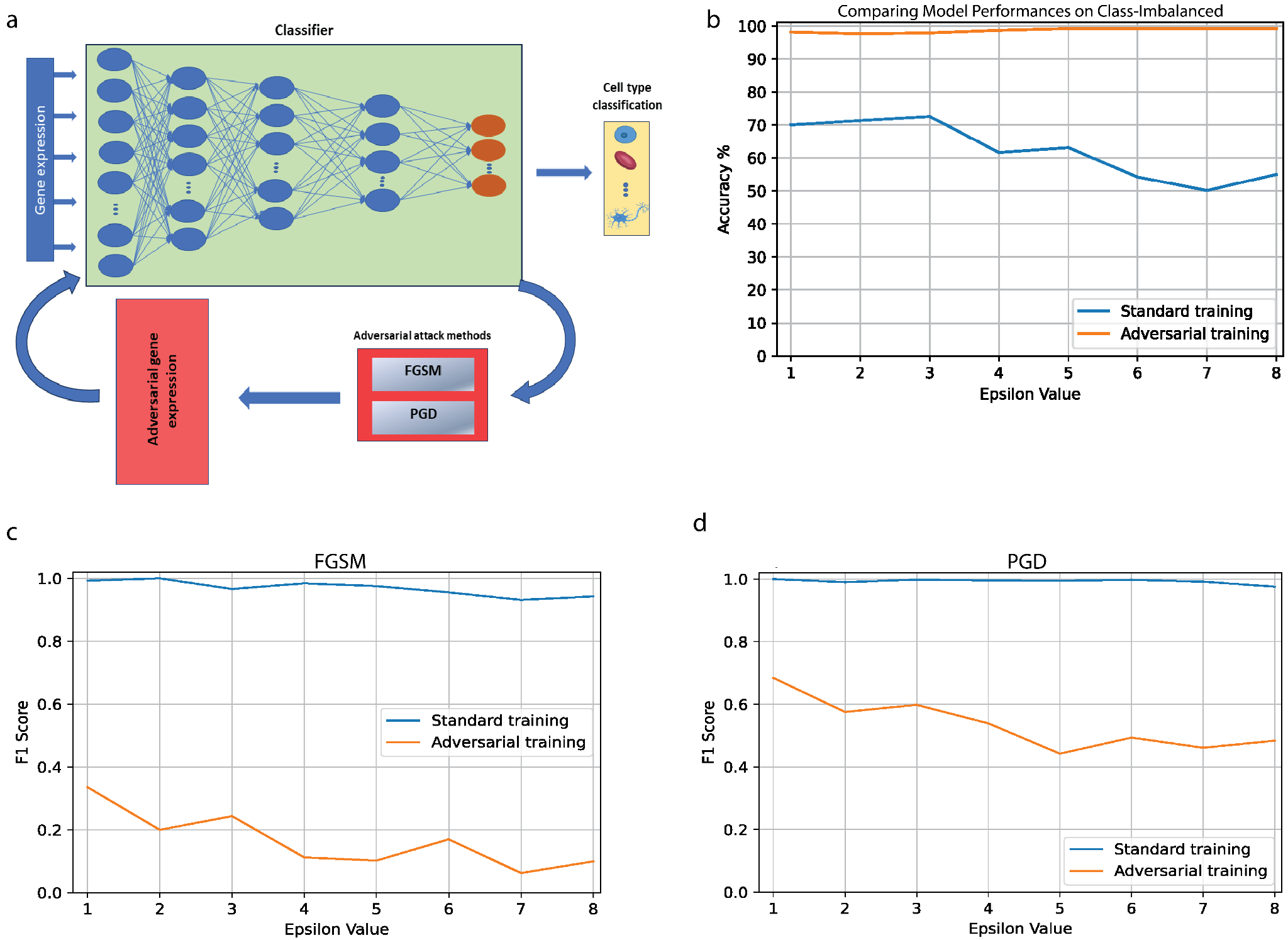


**Supplementary Figure 2 - The pipeline of adversarial training of a multi-layer perceptron for cell type classification. a,** Initially, the neural network is trained using the unperturbed gene expression profile to classify cell types. Then one of the adversarial attack methods is used to generate the adversarial gene expression data which can drastically decrease the performance of the model. The newly created adversarial data is integrated with the original data to train the classifier adversarially. b, By employing FGSM to adversarially train a model using class imbalance data we are able to increase the robustness of the model. The blue line shows the accuracy of the model when tested using perturb data. The orange line shows the performance of the neural network tested on the same perturbed data c,d, Impact of adversarial training on model performance assessed through F1 score after FGSM (c) and PGD attacks (d). Adversarial attacks lead to significant accuracy reduction (indicated by blue lines); however, with proper adversarial training, high accuracy can be regained.


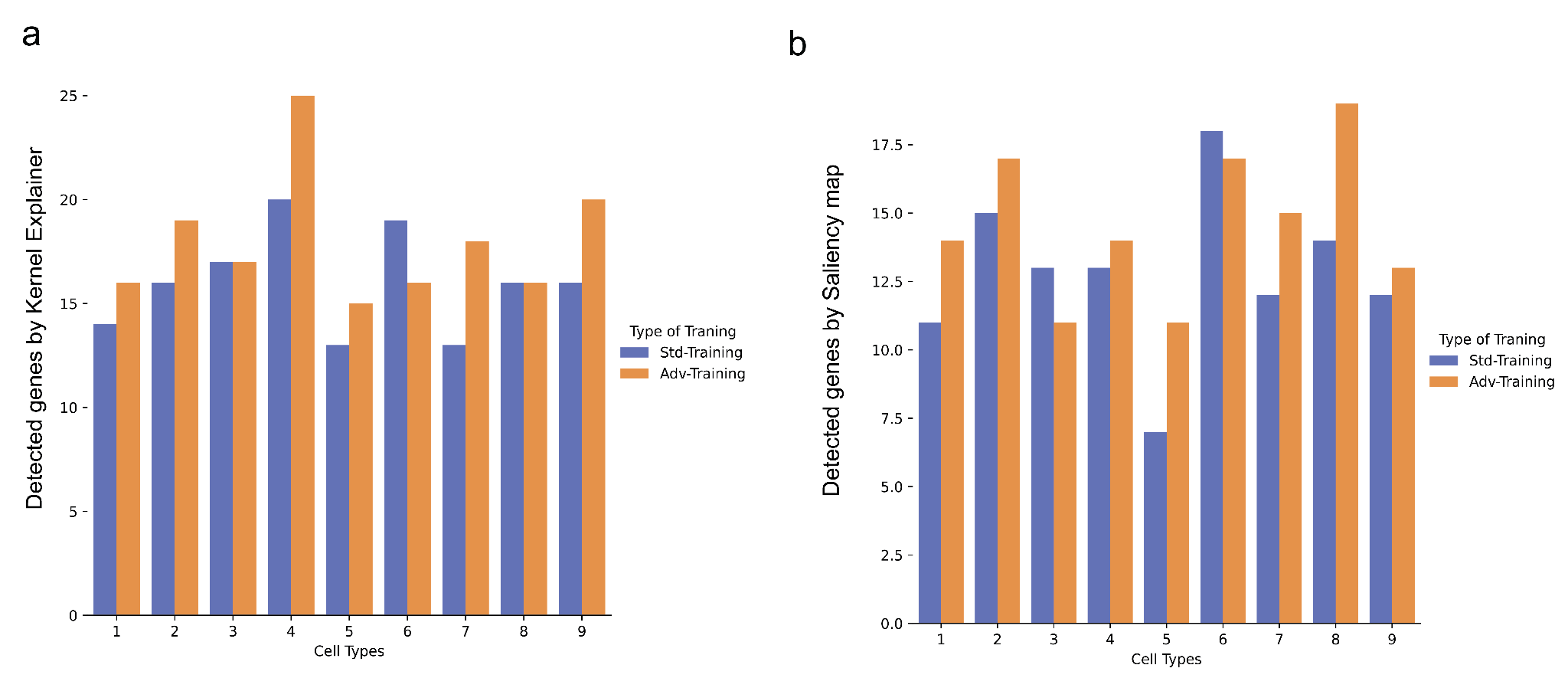


**Supplementary Figure 3- Effect of adversarial training on the model’s interpretability for the simulated data with known key genes.**The effect of adversarial training on the model’s interpretability. a, the results of using the Kernel Explainer; b, Saliency map.


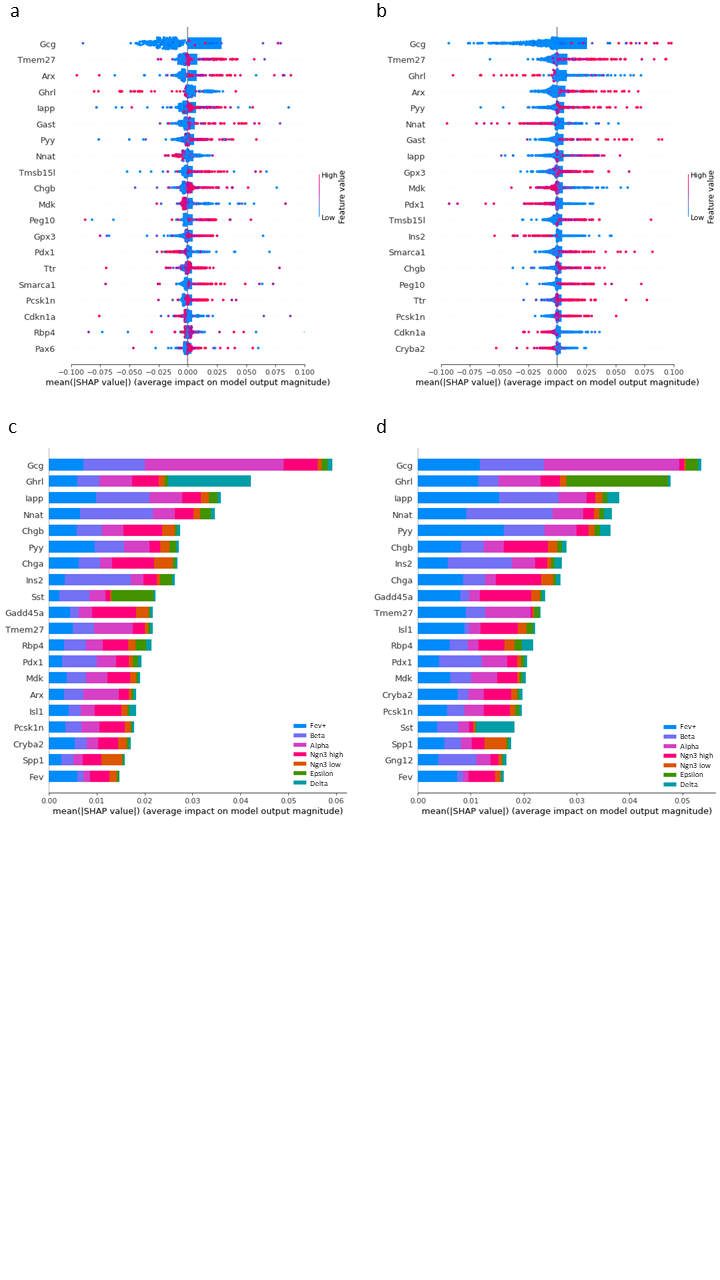
**Supplementary Figure 4: Comparative analysis of different interpretability methods on an adversarially trained model for cell-type classification using mouse Pancreas development data.** (a) and (b) display the top 20 most important genes identified by Deep Explainer and Gradient Explainer, respectively. Each dot represents a gene, and its position on the x-axis corresponds to its SHAP value. (c) and (d) show the mean absolute SHAP values, indicating the rank order of important genes for cell-type classification. These results suggest that different interpretability methods can yield divergent rankings for important genes, highlighting the need for careful consideration of interpretability approaches when analyzing model predictions.


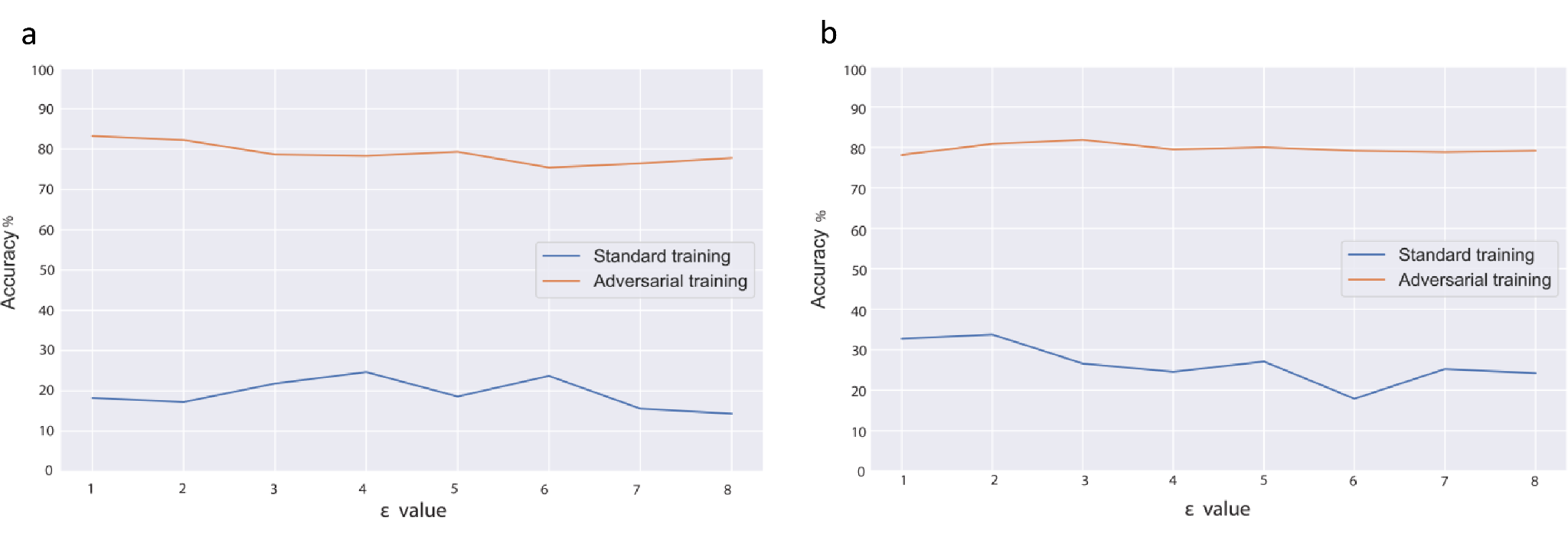


**Supplementary Figure 5: Effect of Adversarial Attack and Training on cell type-classification model Accuracy.**

(a) PGD and (b) FGSM methods demonstrate the impact of adversarial perturbation on model accuracy. The orange curves indicate the performance of models with adversarial training, while the blue curves represent models trained using standard methods. The FGSM and PGD attacks have different strengths, and their effect on model accuracy is apparent in the respective subplots. As can be seen, adversarial training significantly improves the robustness of the models against these attacks.


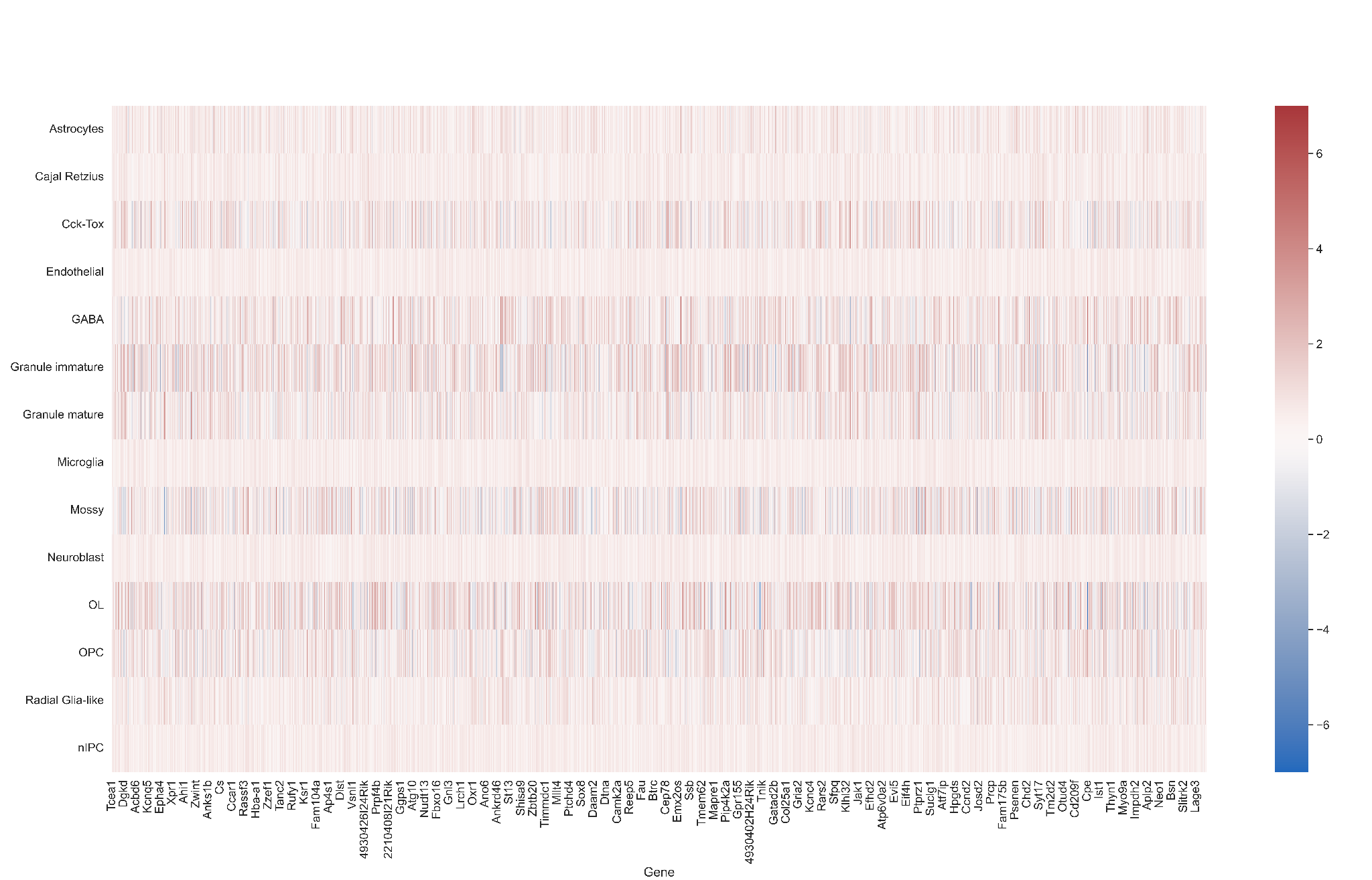


**Supplementary Figure 6 - Activation Maximization scores for each gene-cell type pair for hippocampus development.**

The heatmap of activation maximization scores for gene-cell type pairs. The x-axis corresponds to the genes, while the y-axis represents the cell types. The color of each cell reflects the activation maximization value for the corresponding gene-cell type pair, with red colors indicating higher positive values. This heat map provides a visual representation of the strength and specificity of gene expression across different cell types, which can help in identifying potential biomarkers or therapeutic targets for specific diseases.

| **Name** | **Type** | **Size** | **Activation** |
| --- | --- | --- | --- |
| Layer 1 | Fully connected | 500 | ReLU and Dropout=0.1 |
| Layer 2 | Fully connected | 250 | ReLU |
| Layer 3 | Fully connected | 100 | ReLU |
| Layer 4 | Fully connected | 80 | ReLU |
| Layer 5 | Fully connected | 60 | ReLU and Dropout=0.2 |
| Layer 6 | Fully connected | 40 | ReLU |
| Layer 7 | Fully connected | 20 | ReLU |
| Layer 8 | Fully connected | Number of cell types | Softmax |
| Optimization: Optimizer: Adam, batch_size:500, epochs: 1000 | | | |

**Supplementary table 1 -** Detailed deep neural network architecture for cell-type classification. The same architecture is used for all the datasets we analyze except Allen Brain Map snRNA-seq (Figure 5) where we applied the default parameters of the previously published cell type classification method.
